# Expression of recombinant human Apolipoprotein A-I_Milano_ in *Nicotiana tabacum*

**DOI:** 10.1186/s40643-023-00623-w

**Published:** 2023-01-21

**Authors:** Wei Zhao, Lu-Yang Zhou, Jing Kong, Ze-Hao Huang, Ya-Di Gao, Zhong-Xia Zhang, Yong-Jie Zhou, Ruo-Yu Wu, Hong-Jun Xu, Sheng-Jun An

**Affiliations:** 1 Hebei Provincial Engineering Laboratory of Plant Bioreactor Preparation Technology, Hebei University of Chinese Medicine, No. 326 Xinshi South Road, Shijiazhuang, 050090 Hebei China; 2 School of Nursing of Hebei University of Chinese Medicine, No. 326 Xinshi South Road, Shijiazhuang, 050090 Hebei China

**Keywords:** Apo A-I_Milano_, *N. tabacum*, Recombinant expression, Atherosclerosis, Amino acid sequencing, Turbidity clearance assay

## Abstract

Apolipoprotein A-I_Milano_ (Apo A-I_Milano_) is a natural mutant of Apolipoprotein. It is currently the only protein that can clear arterial wall thrombus deposits and promptly alleviate acute myocardial ischemia. Apo A-I_Milano_ is considered as the most promising therapeutic protein for treating atherosclerotic diseases without obvious toxic or side effects. However, the current biopharmaceutical platforms are not efficient for developing Apo A-I_Milano_. The objectives of this research were to express Apo A-I_Milano_ using the genetic transformation ability of *N. tabacum*. The method is to clone the coding sequence of Apo A-I_Milano_ into the plant binary expression vector pCHF3 with a Flag/His6/GFP tag. The constructed plasmid was transformed into *N. tabacum* by a modified agrobacterium-mediated method, and transformants were selected under antibiotic stress. PCR, RT-qPCR, western blot and co-localization analysis was used to further verify the resistant *N. tabacum*. The stable expression and transient expression of *N. tabacum* were established, and the pure product of Apo A-I_Milano_ was obtained through protein A/G agarose. The results showed that Apo A-I_Milano_ was expressed in *N. tabacum* with a yield of 0.05 mg/g leaf weight and the purity was 90.58% ± 1.65. The obtained Apo A-I_Milano_ protein was subjected to amino acid sequencing. Compared with the theoretical sequence of Apo A-I_Milano_, the amino acid coverage was 86%, it is also found that Cysteine replaces Arginine at position 173, which indicates that Apo A-I_Milano_, a mutant of Apo A-I, is accurately expressed in *N. tabacum*. The purified Apo A-I_Milano_ protein had a lipid binding activity. The established genetic modification *N. tabacum* will provide a cost-effective system for the production of Apo A-I_Milano_. Regarding the rapid propagation of *N. tabacum*, this system provides the possibility of large-scale production and accelerated clinical translation of Apo A-I_Milano_.

**Graphical Abstract:**

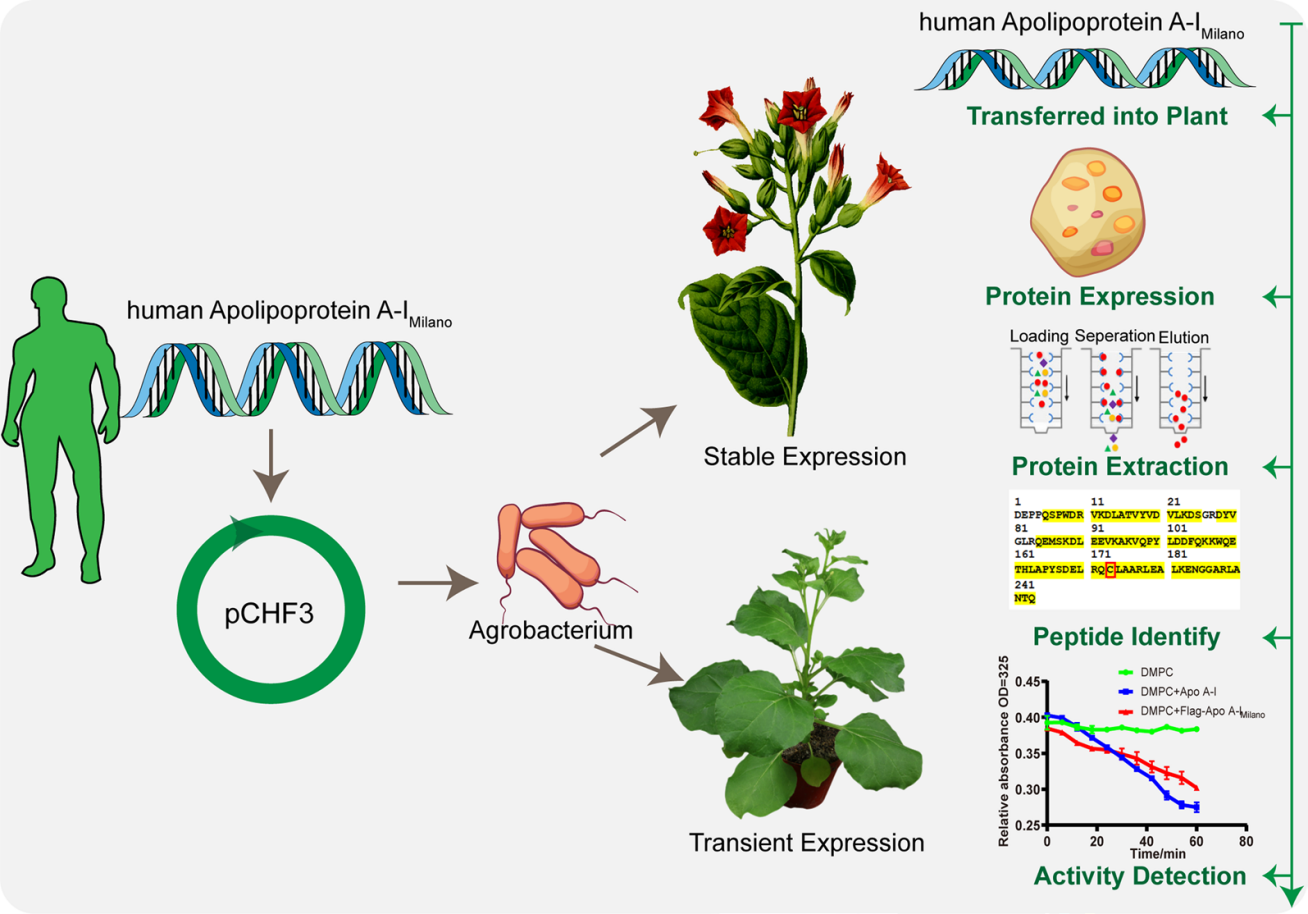

## Introduction

Atherosclerosis can result in coronary and peripheral artery diseases, such as stroke or heart attack. Epidemiological studies have demonstrated an inverse correlation between the levels of high-density lipoprotein (HDL) cholesterol, the so called “good cholesterol,” with the risk of atherosclerosis (Kontush 2020; Chen et al 2020). As the principal component of HDL, Apolipoprotein A-I (Apo A-I) is believed to play an important role in the prevention of atherosclerosis via the process of reverse cholesterol transport (RCT) and anti-inflammatory function (Gaddis et al. 2018; Jackson et al. 2021; Barrett et al. 2019). Normally, low levels of HDL cholesterol would be a high risk of atherosclerosis. However, researchers noticed some inhabitants in an Italian town with low levels of HDL cholesterol but did not affect by atherosclerotic diseases (Franceschini et al. 1980; Weisgraber et al. 1980). Further approach figured out that these subjects expressed a variant of Apo A-I, designated as Apo A-I_Milano_, with its arginine at position 173 replaced with cysteine (Weisgraber et al. 1983).

Since the discovery of the variant Apo A-I_Milano_, researchers have put effort into figure out whether Apo A-I_Milano_ possesses superior atheroprotective effects than the wild type of Apo A-I thus could be developed into a therapeutic. Kaul et al. (Kaul et al. 2004) verified treatment with Apo A-I_Milano_/phospholipid complex could rapidly improve the endothelial dysfunction in hypercholesterolemic Apo E-null mice. Studies on animal injury models (Ibanez et al. 2008; Kaul et al. 2003; Marchesi et al. 2008; Parolini et al. 2008; Speidl et al. 2010) and atherosclerosis patients (Nissen et al. 2003) indicated an infusion of ETC-216 (the complex of recombinant Apo A-I_Milano_ with 1-palmitoyl-2-oleoyl phosphatidylcholine) or its mimetic resulted in plaque regression and reduction of reperfusion injury. Expression of Apo A-I_Milano_ in Apo B/human Apo A-II (h-B/A-II) transgenic mice showed similar atheroprotective features with that expression of Apo A-I gene (L. Wang et al. 2006). However, gene therapy with macrophage-specific expression of Apo A-I_Milano_ exerted a superior effect in the treatment of atherosclerosis in Apo A-I/Apo E double-knockout mice after bone marrow transplantation than Apo A-I (L. Wang et al. 2006). In another study, an infusion of HDL_Milano_ twice with a 4-day interval showed better anti-inflammatory and plaque stabilizing properties than HDL wild type in the treatment of atherosclerotic New Zealand White rabbits (Ibanez et al. 2012). Apo A-I_Milano_ also showed an anti-oxidant activity that distinguished from Apo A-I_wild type_ (Bielicki and Oda 2002). Recently, a report demonstrated that intravenous delivery of human recombinant Apo A-I_Milano_ to the APP23-transgenic mouses reduced their β-amyloid cerebral deposition indicating potential ability to ease Alzheimer (Fernandez-de Retana et al. 2017).

Bioactive experiments and clinical trials require a great amount of Apo A-I_Milano_. Furthermore, considering the prevalent population with arthrosclerosis, application of Apo A-I_Milano_ in the future will also require sufficient Apo A-I_Milano_. Thus, it is essential to develop a cost and capacity efficient manufacturing platform. Recombinant expression of Apo A-I_Milano_ has been achieved in E. coli (Li et al. 2005; Persson et al. 1998; Zhuang et al. 2006), yeast (Zhang et al. 2008). However, copurification of the recombinant Apo A-I_Milano_ with host cell protein is a problem when expressed in E. coli. Purification methods have been optimized to improve the production process (Hunter et al. 2008a, 2007, 2008b; Nord 2000).

Plant-based expression systems are effective for therapeutic protein expression (Fausther-Bovendo et al. 2021; Maharjan et al. 2021; Pillet et al. 2019; Loh et al. 2017). Nykiforuk et al. (Nykiforuk et al. 2011) successfully expressed bioactive Apo A-I_Milano_ in transgenic safflower seeds.

Various expression strategies supply more and more opportunities to the production of pharmaceutical proteins and enzymes of commercial interest in both prokaryotic and eukaryotic species. Among the different expression platforms involving different organisms, plants have long been potential taken as an attractive platform for the production of unlimited number of recombinant proteins, including pharmaceutical proteins, such as monoclonal antibodies, vaccines, and enzymes. The first recombinant plant-derived pharmaceutical protein proved to be human serum albumin expressed in transgenic *N. tabacum* and potato plants in 1990 (Sijmons et al. 1990). Compared to traditional approaches in molecular farming of pharmaceuticals, the plant expression systems obviously showed advantages including their low costs, particularly when considering large-scale production. Microbial and animal cell cultures require specific equipment and electric energy supply, while plants can synthesize any protein and metabolite from CO_2_ and inorganic chemicals using solar energy. In addition, the limited risks of contamination by viruses or pathogens can be minimized. Moreover, protein purification can be eliminated when suitable plant tissue containing recombinant protein is used as food, such as lettuce leaves, tomato fruit. Importantly, plants also displayed capable of conducting complex post-translational modifications required for recombinant pharmaceutical proteins, including N-glycosylation, which is substantially similar to that found in mammalian cells. Many plant species have been tested for their ability to produce recombinant pharmaceutical proteins, including Nicotiana species, safflower, tomato, potato, soybean, alfalfa, spinach, A. thaliana, corn, and rice. *N. tabacum* is one of the ideal expression systems in plant bioreactor based on several practical advantages over other crops. It produces significant leaf biomass (up to 100 t of leaf biomass per hectare), has high soluble protein content and is a non-food crop. In addition, various methods of protein expression could be carried out in *N. tabacum*, including transient or stable expression via the agrobacterium. So far, it has been reported that stable nuclear transformation in *Nicotiana tabacum* has commonly been used as an excellent production platform of some therapeutic antibodies (Sack et al. 2015; Buyel et al. 2017). More importantly, the stable transformation of transgenic *N. tabacum* requires neither costly fermenters, nor vacuum infiltration equipment, nor sterile conditions. Although *N. tabacum* taken as an ideal plant for the production of medicinal proteins has many advantages, yet several challenges need to be addressed to achieve comparable efficiency as the mammalian system. The relatively low expression frequency was one of the main challenges in promoting *N. tabacum* is a significant recombinant protein production system. So far, there is still a lack of *N. tabacum* culture system for the expression of Apo A-I_Milano_.

In order to explore the method of producing Apo A-I_Milano_ in the plant reactor, in this study, the model plant *N. tabacum* tissue culture technology and Agrobacterium mediated genetic transformation were used to obtain transformed plants. The surviving transformed plants were verified by PCR and RT-PCR technology. The obtained positive plants were harvested seeds and then continued to be planted, propagated and identified to obtain a stable genetic *N. tabacum* line. Meanwhile, the expression, subcellular localization and purification of Apo A-I_Milano_ protein in *N. tabacum* were completed by transient transformation, fluorescence labeling and chromatography. This study was the first to report the transient and stable expression of Apo A-I_Milano_ protein in *N. tabacum*.

## Materials and methods

### Construction of plant expression Vector pCHF3–Flag–Apo A-I_Milano_, pCHF3–Apo A-I_Milano_–GFP, pCHF3–GFP, and pCHF3–His6tag–GFP–TEV–Apo A-I_Milano_

**Fig. 1:**
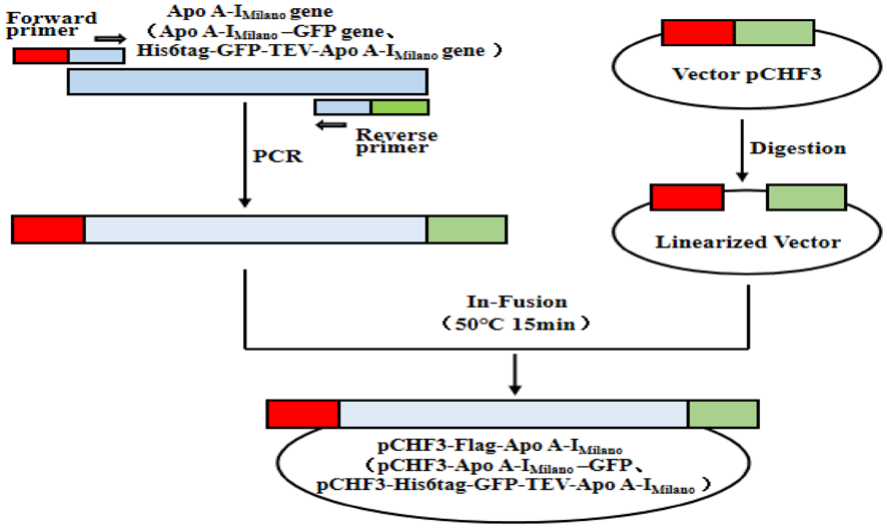
Construction of recombinant plasmid pCHF3–Flag–Apo A-I_Milano,_ recombinant plasmid pCHF3–Apo A-I_Milano_–GFP and pCHF3–His6tag–GFP–TEV–Apo A-I_Milano_. The plant expression vector pCHF3, encoding a fusion protein consisting of 3 × Flag (GFP, His6tag–GFP) was fused in-frame with Apo A-I_Milano_. The coding sequence was under the transcriptional control of a CaMV 35S promoter

The DNA of the Apo A-I_Milano_ gene was synthesized by GENEWIZ Company (GENEWIZ, China). To facilitate the detection of the target protein by western blot, a 3 × Flag tag was added to the N-terminus of the recombinant protein. Phanta Max Master Mix PCR kit (Cat. No. P525, Vazyme, China) was used and PCR was performed using gene-specific primers (forward 5’-CGGGGGACGAGCTCGGTACCATGGTTAACGACTACAAAGACGATGACGACAAGGACTACAAAGACGATGACGACAAGGACTACAAAGACGATGACGACAAGGATGAGCCTCCTCAATC-3’and reverse 5’-GCAGGTCGACTCTAGATCATTGAGTATTAAGCTTCTT-3’) by the following protocol: 98 °C for 2 min, followed by 35 cycles of amplification (94 °C for 40 s, 58 °C for 30 s, 72 °C for 30 s, with the final elongation step at 72 °C for 5 min.). The PCR product was purified with a Gel Extraction Kit (Transgenes, China), then cloned into the pCHF3 vector (kindly provided by Tobacco Research Institute of Chinese Academy of Agricultural Sciences) using ClonExpress^®^ Ultra One Step Cloning Kit (Vazyme, China). The empty pCHF3 was digested with KpnI and XbaI to obtain pCHF3–Flag–Apo A-I_Milano_. The recombinant constructs were transferred into E. coli DH5α competent cells. Grown colonies were detected by the PCR method using the specific forward and reverse primers (forward 5’-GCAAGTGGATTGATGTGATAT-3’ and reverse 5’-TAAGCTTCTTAGTATATTCTTC-3’). Then, the clone was sequenced to confirm the correct sequence, which is a 934 bp long fusion gene of vector, 3xFlag and Apo A-I_Milano_. The gene Apo A-I_Milano_ was driven by the control of the strong cauliflower mosaic virus (CaMV) 35S promoter in pCHF3. Then, the construction was transformed into Agrobacterium tumefaciens (A.tumefaciens) strain GV3101 (Biomed, China) using freeze-thaw method (Fig. 1).

To further detect the distribution and localization of the target protein in tissues and cells, we also constructed a plasmid expressing pCHF3–Apo A-I_Milano_–GFP fusion protein and took pCHF3–GFP as the control. Simultaneously, pCHF3–his6tag–GFP–TEV–Apo A-I_Milano_ plasmid was also constructed for the purification of the target protein Apo A-I_Milano_ and its amino acid sequence determination and analysis. The construction method was the same as that of pCHF3–Flag–Apo A-I_Milano_. The recombinant plasmid was cloned and identified by GENEWIZ Company (Fig. 1).

#### Preparation of agrobacterium strains harboring pCHF3–Flag–Apo A-I_Milano_ for infiltration

To prepare the appropriate scale A. tumefaciens, GV3101 harboring pCHF3–Flag–Apo A-I_Milano_ was cultured in 50 ml of YEB medium supplemented with 100 µg/ml of spectinomycin and 50 µg/ml of rifampicin. Then, the cultures were incubated at 28 ℃ in 230 rpm constant shaking condition overnight. To prepare infiltration buffer, the *A. tumefaciens* culture mentioned above were harvested by centrifugation at 6000 rpm for 5 min at room temperature, then, prepared infiltration buffer (10 mM MES, 150 μM AS, 10 mM MgCl_2_) dissolved the suspension and the OD_600_ was adjusted to 0.8 to 1.0. Then, the culture was incubated at room temperature without any agitation for at least 3 h before infiltration. Preparation of A. tumefaciens GV3101 for pCHF3–Apo A-I_Milano_–GFP and pCHF3–GFP was as same with pCHF3–Flag–Apo A-I_Milano_.

#### The growth conditions of *N. tabacum* and preparation of *N. tabacum* leaves disks for stable transformation

Seeds of *N. tabacum* were obtained from China National GeneBank (ID: CNSebb2006170), Seeds of Hong Hua Da Jin Yuan (HD) were obtained from TRI of the Chinese Academy of Agricultural Sciences (ID: HD), originally donated by the Chinese Academy of Agricultural Sciences for collection of seeds. Seeds were surface-sterilized using 75% alcohol for 1 min 30 s and 10% sodium hypochlorite for 15 min followed by washing with autoclaved distilled water 3 times. The seeds were then grown on Murashige and Skoog (MS) medium. The pots were placed in a growth chamber under controlled conditions of 25–30 ℃with 16 h light/8 h dark photoperiod. All plant materials used in this experimental study abide by the national safety implementation measures and management regulations in the process of planting, transformation, sampling and testing, these regulations include “Safety Administration Implementation Regulation on Agricultural Biological Genetic Engineering” and “Tobacco and Tobacco Products- Detecting Method of Genetically Modified Organism Contents (GB/T 24,310–2009)”.

The transformation of *N. tabacum* was performed by co-cultivation as described previously (Tang et al. 2005). The explants were subcultured in different mediums for shoot induction and root induction. Briefly, the leaf discs of 1 cm in diameter were prepared from the cultivation seedlings and incubated for 10 min in A. tumefacien solution (OD_600_ = 0.6–0.8). The leaf discs were then blotted onto filter paper to remove excess bacterial suspension. The infected leaves were plated on the co-cultivation medium (MS with 1 mg/L 6-BA, 0.1 mg/L IAA) with the veins facing up, and cultured in the dark at 25 °C for 3 days. Then, leaf discs were placed upside down on S1 medium (MS with 1 mg/L 6-BA, 0.1 mg/L IAA, 500 mg/L cefotaxime sodium and 50 mg/L kanamycin) for 2–3 weeks. Then, the leaf discs were transferred to S2 medium (MS with 0.5 mg/L 6-BA, 0.05 mg/L IAA, 500 mg/L cefotaxime sodium, and 50 mg/L kanamycin) for 1–2 weeks. Then, seeding grow from the callus was then transferred onto S3 medium (MS with 0.5 mg/L 6-BA, 0.02 mg/L IAA, 500 mg/L cefotaxime sodium and 50 mg/L kanamycin) for 1–2 weeks and then transferred onto R medium (MS with 500 mg/L cefotaxime sodium and 50 mg/L kanamycin) until the root was detected. All tissue culture experiments were conducted in a growth chamber at 25 ℃ and a photoperiod of 16 h/8 h day/night. The well-rooted transgenic plants were transferred to soil under a controlled photoperiod of 16 h light/8 h dark at 25 ℃.

**Fig. 2:**
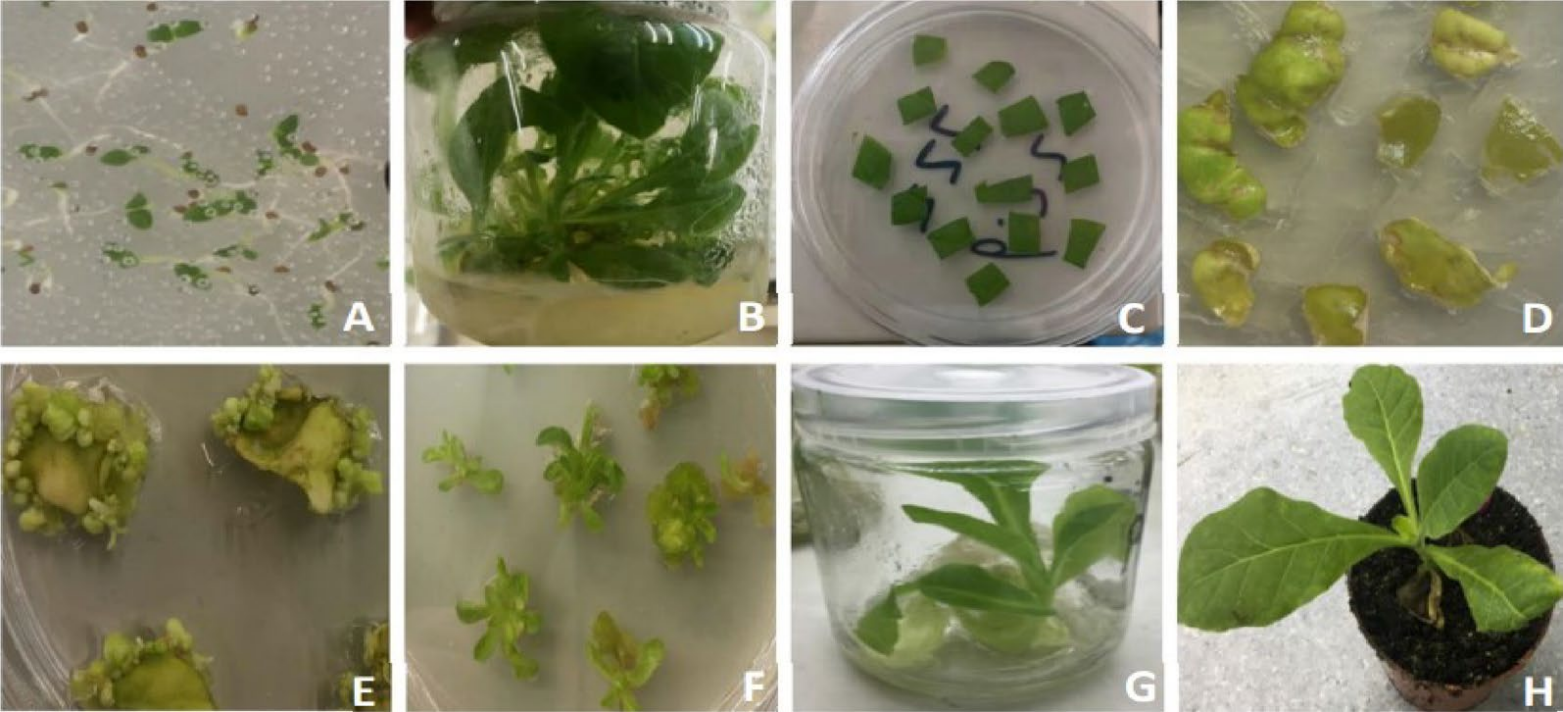
Regeneration of transgenic *N. tabacum* plants. **A** Sterilized seed laying board. **B** Aseptic seedlings for cutting leaves. **C** Co-culture of leaves and Agrobacterium tumefaciens. **D** Infected leaves. **E** Callus differentiation. **F** *N. tabacum* regenerated seedlings. **G** Rooting. **H** *N. tabacum* seedlings have moved into the soil

*N. tabacum* seeds were sterilized with 75% alcohol for 1 min and 30 s and 10% sodium hypochlorite for 15 min followed by washing with autoclaved distilled water 3 times. After disinfection, sow seeds into MS medium (Fig. 2A), and moved the seedlings were to a tissue culture flask when they grew to about 0.5 cm; when the number of leaves reached about 8 leaves (Fig. 2B), green leaves were selected for agrobacterium infection. pCHF3–Flag–Apo A-I_Milano_ was transferred into *N. tabacum* leaves using agrobacterium-mediated method and co-cultured for 3 days (Fig. 2C). The co-cultured leaves were then inoculated on S1 medium (Fig. 2D), and the generation of tufted buds could be seen about 2 weeks later (Fig. 2E). The tuft buds in S1 medium were transferred to S2 and S3 medium, and after about 2 weeks of culture, the tuft buds grew into young seedlings (Fig. 2F). When the resistant seedlings grew to about 3 cm, small seedlings were cut and transferred to R medium to induce rooting, and roots were generated and seedlings gradually formed about 2 weeks later (Fig. 2G). After the seedlings were grown, the seedlings were transplanted into the flowerpots in the greenhouse (Fig. 2H).

#### Molecular characterization of stable transgenic *N. tabacum*

Genomic DNA was extracted from the leaves of putative transgenic *N.* *tabacum* lines using a Plant DNA extraction Kit (CWbio Inc., China) following manufacture’s protocol. PCR analyses were performed using primer sequences (forward 5’-GCAAGTGGATTGATGTGATAT-3’ and reverse 5’-TAAGCTTCTTAGTATATTCTTC-3) to identify positive transgenic plants. The cycling schedule of PCR was 95 ℃ for 10 min; 30 cycles of 95 ℃ for 1 min, 60 ℃ for 1 min, and 72 ℃ for 50 s, with a final extension at 72 ℃ for 10 min. PCR products were electrophoresed on 1.5% agarose gel, then stained with ethidium bromide and visualized under UV light. The amplified DNA fragment including vector, 3xFlag and Apo A-I_Milano_ was 934 bp.

For fluorescence quantitative analysis of transgenic *N.* *tabacum*, total RNA from *N.* *t tabacum* leaves was extracted after quick-freezing in liquid nitrogen. The cDNA was reversely transcribed (PrimeScriptTM RT reagent Kit with gDNA Eraser, Code No. RR047A, Takara, Japan) and analyzed by fluorescent quantitative PCR. Actin (NT-L25) was selected as the reference gene, and the primer sequence was as follows: Actin-F: GCTAAGGTTGCCAAGGCTGTC; Actin-R: TAAGGTATTGACTTTCTTTGTCTGA; The PCR primer sequence of the Apo A-I_Milano_ target gene was F: AGCCTCCTCAATCTCCTTGG; R: TTGCTTACCAAGAGCAGAACCT. Total RNA was extracted from stable transgenic and non-transgenic *N. tabacum* leaves tissues using the RNA extraction kit (Transgene, China) according to the manufacturer’s instruction. First-strand cDNA was synthesized after genomic DNA was eliminated by DNase I. RT-qPCR was performed using the following first-strand cDNA as template using the procedure: 95 °C for 300 s; 40 cycles of 95 °C for 10S, 60 °C for 30S; 95 ℃ for 15 s, 60 ℃ for 60 s.

For Western blotting of the stable transformation, proteins were extracted from the transformation and non-transformed leaves of *N. tabacum* using lysis buffer (Thermo, USA) and protease inhibitor. The samples were centrifuged at 14,000 × g for 15 min before loading on 4% stacking and 12% separating SDS–polyacrylamide gel (SDS–PAGE) after boiling at 100 °C for 10 min. The mouse monoclonal antibody against human Apo A-I (Santa Cruz, USA) was used as the primary antibody. The antibody was diluted to 300-fold and used to incubate the electrophoretically separated protein extract and the electroimprinted membrane. The goat anti mouse (Proteintech, USA) antibody diluted 5000-fold was used as the second antibody.

#### Infiltration of *N. tabacum* using a syringe for transient transformation

**Fig. 3:**
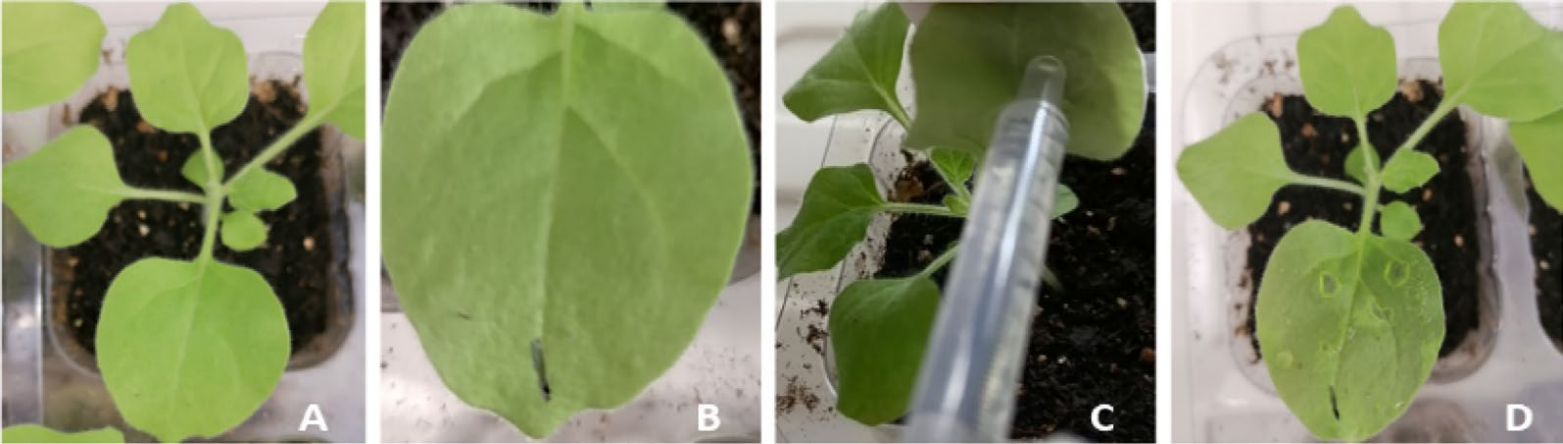
Transient expression in *N. tabacum*. **A** Selected *N. tabacum* plants with good growth conditions. **B** Marked *N. tabacum* leaves. **C** Injected Agrobacterium containing pCHF3–Flag–Apo A-I_Milano_ with an OD_600_ of 0.8–1.0 from the lower epidermis of *N. tabacum* leaves with a 1 ml syringe with the tip removed. **D** Injection completed

*N.* *tabacum* grown under constant light conditions for 4 weeks in a greenhouse was taken to infiltrate using syringe according to the described by Abd-Aziz, N. et al. (Abd-Aziz et al. 2020). Briefly, the infiltration buffer with OD_600_ of 0.8–1.0 containing *A. tumefaciens* strain GV3101 harboring pCHF3–Flag–Apo A-I_Milano_ synthesized by GENEWIZ Company were respective injected into the leaf with a syringe without a needle. Then, the plants were cultured in a 24 h dark condition. At least 3 days post-infiltration culture before the following treatment including analysis of mRNA and protein expression (Fig. 3).

#### RT-PCR and western blotting of transient transformation

Total RNA was extracted from transient transgenic and non-transgenic *N. tabacum* leaves tissues using the RNA extraction kit (Transgene, China) according to the manufacturer’s instruction. First-strand cDNA was synthesized after genomic DNA was eliminated by DNase I. PCR kit (TB Green^®^ Premix Ex Taq^™^, Code No.: RR420A, Takara, Japan) was used and PCR was performed using the following first-strand cDNA as a template using the procedure: 95 °C for 300 s; 30 cycles of 95 °C for 15S, 45 °C for 30S, and 72 °C for 60S; and 72 °C for 300 s for a final extension. The amplified PCR products were analyzed by 1% TAE Agarose gel. (Forward 5’-ATGGTTAACGACTACAAAGACG-3’ and reverse 5’-TCATTGAGTATTAAGCTTCTTAGT-3’).

For Western blotting, the steps were the same as those for stable transgenic *N.* *tabacum*.

#### Subcellular localization of target protein in *Nicotiana benthamiana*

Seeds of *Nicotiana benthamiana (N. benthamiana)* were obtained from China National GeneBank (ID: CNS0440294), *N. benthamiana* plants grown in a growth chamber under controlled conditions of 25–30 ℃, 70% relative humidity with 16 h light/8 h dark photoperiod. All plant materials used in this experimental study abide by “Safety Administration Implementation Regulation on Agricultural Biological Genetic Engineering” and “Tobacco and Tobacco Products- Detecting Method of Genetically Modified Organism Contents (GB/T 24,310–2009)”.

GV3101 containing pCHF3–Apo A-I_Milano_–GFP, pCHF3–GFP, and ER marker plasmid were, respectively, grafted into 10 ml YEB liquid medium (yeast extract 4.0 g/L, mannitol 10.0 g/L, NaCl 0.1 g/L, MgSO_4_ 0.2 g/L, K_2_HPO_4_ 0.5 g/L, pH = 7.0) and cultured at 170 rpm for 1 h. Then, the supernatants were removed and collected by centrifugation at 4000 rpm for 4 min. The bacteria were re-suspended with 10 mM MgCl_2_ (with 120 μM AS) suspension and OD_600_ was adjusted to about 0.6. *N. tabacum* plants with good growth conditions were selected, and agrobacterium containing marker plasmids and agrobacterium containing pCHF3–Apo A-I_Milano_–GFP/pCHF3–GFP vector plasmids were suspended together for the operation. The endoplasmic reticulum (ER) localization signal protein was Sper, its amino acid sequence was MKTNLFLFLFLIFSLLLSLSSAEF. The mixture was mixed in a ratio of 1:1, and injected from the lower epidermis of *N. benthamiana* leaves with a 1 ml syringe without the spear head and made notes. The injected *N. benthamiana* plants were cultured under low light for 2d, and the *N. benthamiana* leaves injected with labeled agrobacterium tumefaciens were made into glass slides, which were observed under a laser confocal microscope (Nikon, Japan) and photographed. The Sper excitation light was 561 nm and the emitting light was 580 nm. Chloroplast fluorescence signal excitation wavelength was 640 nm and the emission wavelength was 675 nm.

#### Purification of expressed target proteins from transient transformation

When the GV3101 Agrobacterium with the target gene had an OD_600_ value of 0.8–1.0, let it stand for 3 h at room temperature. After the standstill was completed, the *N. tabacum* leaves in good condition were injected with a 1 ml needleless syringe. After the injection was completed, culture was in the greenhouse for 72 h for the sample. Put 40 fresh leaves (7 g) into liquid nitrogen and ground to powder, add a lysis buffer (Thermo, USA) with protease inhibitor to the powder on ice; then centrifuged for 15 min to take the supernatant, and added Flag antibody (Sigma-Aldrich, USA) to mix overnight at 4 °C. After that added protein A/G (Thermo, USA) to the supernatant, mixed for 3 h at 4 °C, the samples were centrifuged at 800 × g. Then collected protein A/G and washed them with 1 × PBS. After 3 times, the protein was eluted with Tris–HCl (PH = 7.4). Diluted a portion of the eluted protein by 10 times was used for the BCA protein concentration determination.

### Determination of protein purity by SDS–PAGE

This experiment was conducted by protein purity determination SDS–PAGE method according to 《Guide to Protein purification》(Second Edition, Edited by Richard R. Burgess and Murray P. Deutscher. 2009. Elsevier Inc.). In brief, the purity of purified Apo A-I_Milano_ and Flag fusion protein was detected by SDS–PAGE gel staining. The purified protein solution is subjected to SDS–PAGE. Followed by Coomassie brilliant blue staining and then decolorized to enter the automatic gel imaging system (Tanon-3500R, Shanghai Tanon Technology Co., Ltd., China) for exposure using white light source to obtain gel images. and the image is saved as a TIFF file. ImageJ (NIH) is used to quantify the grayscale of the purified protein in the gel image (Alonso Villela SM et al. 2020), and the ratio of the purified Flag–Apo A-I_Milano_ to the total protein was obtained, which was calculated as the purity of the purified protein.

### The amino acid sequence of Apo A-I_Milano_ in *N. tabacum* was analyzed by mass spectrometry

The fusion protein produced according to step 2.5 was purified with a His protein purification kit (Thermo, USA), and then the amino acid sequence of the fusion protein was analyzed according to the following steps: the protein solution was reduced with 2 µl 0.5 M Tris (2-carboxyethyl) phosphine (TCEP) (Sigma, USA) at 37 °C for 60 min and alkylated with 4 µl 1 M iodoacetamide (IAM) at room temperature for 40 min in darkness. Five folds volumes of cold acetone (Sinopharm, China) were added to precipitate protein at − 20 °C overnight. After centrifugation at 12000 g at 4 °C for 20 min, the pellet was washed twice by 1 ml pre-chilled 90% acetone aqueous solution. Then, the pellet was re-suspended with 100 µl 10 mM Triethylammonium bicarbonate (TEAB) (Sigma, USA) buffer. Trypsin (Promega, USA) was added at 1:50 trypsin-to-protein mass ratio and incubated at 37 °C overnight. The peptide mixture was desalted by C18 ZipTip (Shimadzu Corporation, 5010–21,701, Japan), and lyophilized by SpeedVac (Thermo Scientific, Savant SPD1010, USA). The sequence of the fusion protein was confirmed at Qingdao Sci-tech Innovation Quality Testing Co. Ltd.

### Activity detection of target proteins

Dimyristoyl Phosphatidylcholine (DMPC) dry powder was suspended in TBS (PH = 7.4, 3.5 mg/mL) at a concentration of 1.2 mg/ml. It oscillated violently on the vortex oscillator for 3–5 min to form multilayer liposomes. The purified protein sample was diluted to 0.17 mg/ml. The 200 µl target protein samples and 50 µL of DMPC liposome were incubated in 24 ℃ water baths for 10 min. Total divided into three groups: negative control group DMPC + TBS (PH = 7.4, 3.5 mg/ml); DMPC + purified target protein in the experimental group; Positive control group DMPC plus standard substance (in this experiment, the mass ratio of Apolipoprotein to DMPC liposome was 1:2, to be exact, Apolipoprotein final concentration: 0.12 mg/ml; DMPC liposome final concentration: 0.24 mg/ml). The absorbance value at 325 nm was measured at room temperature, every 2 min, and monitored for 60 min until the absorbance value stabilized. The decrease in absorbance of three independent samples ± SD was plotted over time.

### Statistical analysis

All data are expressed as mean ± standard deviation, the mean comparison between the two groups was performed by *t* test, and a two-tailed test P < 0.05 was considered statistically significant.

## Results

### Introduction of exogenous genes and identification in stable transgenic *N. tabacum*

#### Regeneration and identification of stable transgenic *N. tabacum*

**Fig. 4:**
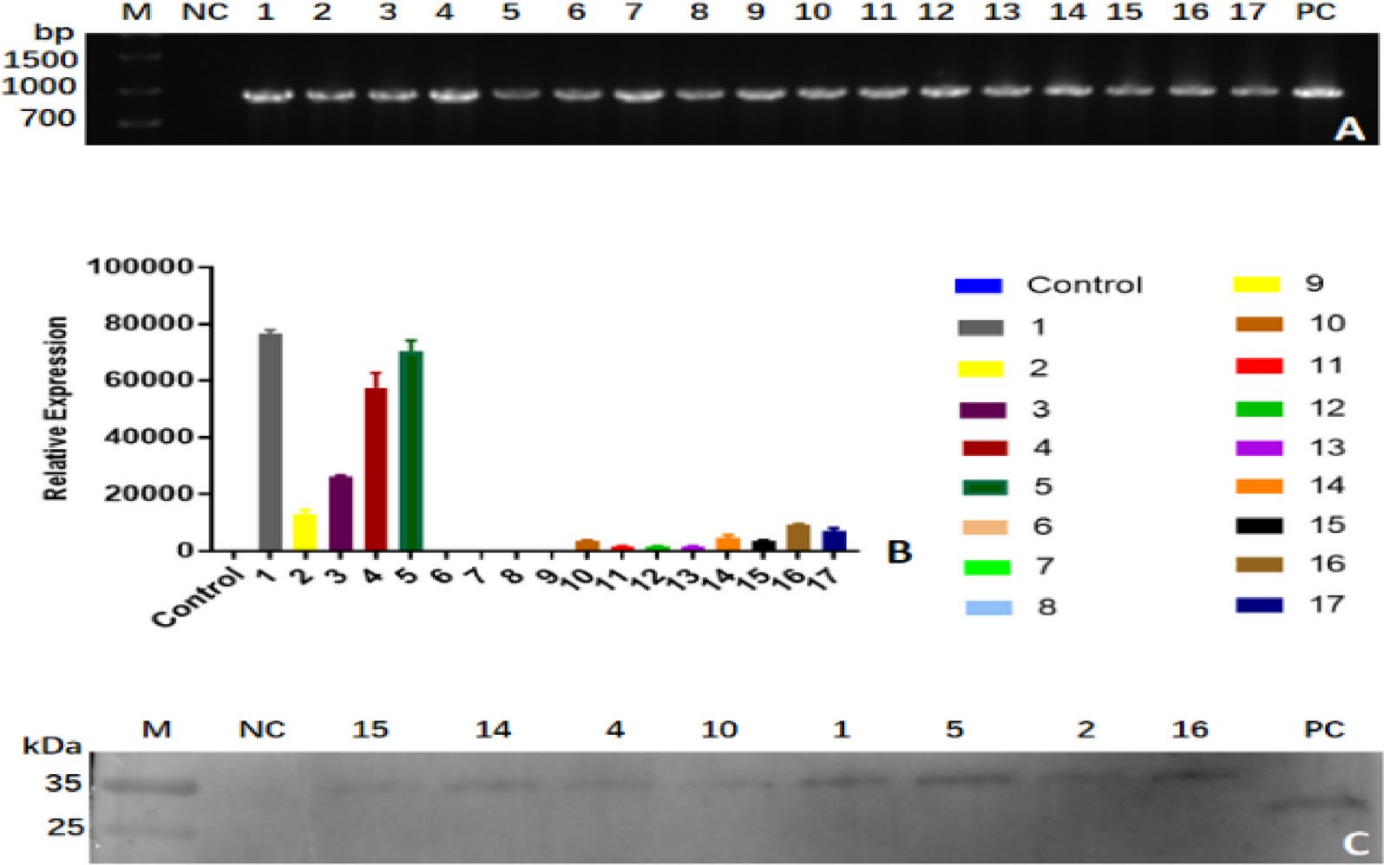
**A** PCR confirmation of the presence of the Apo A-I_Milano_ gene and 3xFlag (934 bp) in stable transgenic *N. tabacum* leaves. Lane M is 2000 bp DNA ladder. Lane NC is the negative control. Lanes 1 to 17 are the PCR products of antibiotic-resistant *N. tabacum* plants, p478 represents the batch number of the stable transgenic *N. tabacum* plants, and numbers 1 to 17 in the PCR test results correspond to p478-9, p478-35, p478-39, p478-53, p478-63, p478-64, p478-67, p478-69, p478-73, p478-7, p478-13, p478-14, p478-21, p478-32, p478-51, p478-56, and p478-74 plants, respectively, These plants are the number of 17 Apo A-I_Milano_ DNA-positive individual plants selected from 86 transgenic *N. tabacum* plants in the same batch p478. Lane PC was a positive control. **B** RT-qPCR analysis of stably transgenic *N. tabacum* leaves from 13 plants with positive mRNA expression were screened from 17 plants with positive DNA detection in the PCR test results. These were p478-9, p478-35, p478-39, p478-53, p478-63, p478-7, p478-13, p478-14, p478-21, p478-32, p478-51, p478-56, p478-74 plants. **C** Western blotting of transformants. Lane M is protein molecular weight marker. Lane NC presents wild *N. tabacum*. Lanes 2 to 9 present recombinant fusion expression of Flag–Apo A-I_Milano_ in stable transgenic *N. tabacum* with a molecular weight of 30 KDa. Lane PC presents the standard protein Apo A-I with a molecular weight of 28 KDa. Numbers 2 to 9 in the western blot results correspond to numbers of p478-51, p478-32, p478-53, p478-7, p478-9, p478-63, p478-35, p478-56 plants, respectively. These are 8 plants with positive expression of Apo A-I_Milano_ protein selected from 13 plants with positive mRNA expression of *N. tabacum*

Considering that *N. tabacum* leaves could produce high biomass, we carried out Apo A-I_Milano_ ectopically overexpressed in *N. tabacum* using the binary vector pCHF3, in which the target gene was driven by 35CaMV. 17 putative transgenic plants were obtained from explant using antibiotic resistance selection. Genomic DNA was used as the template, PCR using gene-specific primers was carried out to detect the T-DNA insertion; the results demonstrated that the target band (Fig. 4A) was observed in the transgenic strain but not wild types, indicating stable Apo A-I_Milano_ gene integration into the genome of the *N. tabacum*.

#### mRNA expression in stable transgenic *N. tabacum*

Fluorescence quantitative PCR was used to analyze the expression levels of target genes in stable transgenic *N. tabacum*. The reference gene NT-L25 was used for correction and standardization. The results showed the relative expression level of P478 batch *N. tabacum* (Fig. 4B), which was used for further detection and analysis.

#### Protein detection in stable genetic *N. tabacum*

SDS–PAGE was used to analyze the protein in the leaves of stable transgenic *N. tabacum*. Compared with the wild-type *N. tabacum* leaves, the expected band appeared at 30 KDa in transgenic P478 batch *N. tabacum* leaves, whereas no obvious band was found in wild-type lines. The expected band appeared at 28 KDa in the positive control, as shown in Fig. 4C. It can be preliminarily proved that Apo A-I_Milano_ was expressed in *N. tabacum* leaves.

### Transient transformation and analysis of Apo AI_Milano_ in *N. tabacum*

#### mRNA expression in transient transgenic *N. tabacum*

**Fig. 5:**
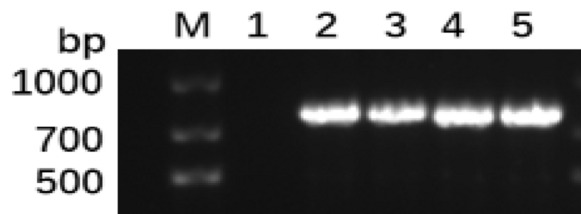
RT-PCR analysis of transformants. Lane M is 2000 bp DNA ladder. Lane 1 is a negative control. Lanes 2–4 are RT-PCR products of transformants after transient transformation for 3 days after injection. Lane 5 is a positive control

To determine whether there is Apo A-I_Milano_ transcription in transgenic *N. tabacum*, the expression of Apo A-I_Milano_ in transgenic *N. tabacum* was detected by RT-PCR. As can be seen from Fig. 5, RT-PCR analysis verified that these *N. tabacum* were positive for transgenic *N. tabacum*.

#### Transient expression in *N. tabacum* Apo A-I_Milano_ produced

**Fig. 6:**
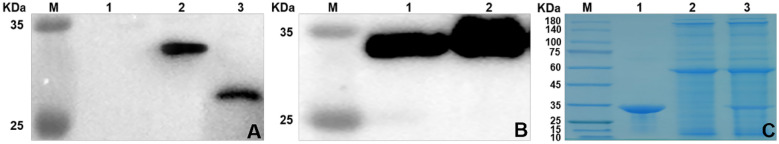
**A** Western blotting of transformants. Lane M is protein molecular weight marker. Lane 1 presents wild *N. tabacum*. Lane 2 presents recombinant fusion expression of Apo A-I_Milano_ and Flag (Flag–Apo A-I_Milano_) with a molecular weight of 30 KDa in transient transgenic *N. tabacum*. Lane 3 shows the expression of the Apo A-I standard protein with a molecular weight of 28 KDa. **B** Western blot analyser of protein purification. Lane M is protein molecular weight marker. Lane 1 presents total protein recombined expression of Apo A-I_Milano_ and the Flag proposed before purification. Lane 2 presents purified Flag–Apo A-I_Milano_ protein. **C** Coomassie blue staining of recombinant Flag–Apo A-I_Milano_ proteins in SDS–PAGE. Lane M is standard protein marker. Lane 1 presents Flag–Apo A-I_Milano_ protein purified by 12.5% SDS–PAGE gel. Lane 2 presents the liquid that flows out of the extracted total leaf protein after passing through Protein A/G column, namely, the efflux liquid. Lane 3 presents total leaf protein

Transient expression could provide fast protein expression within 3–5 days, which confers this strategy overcome some drawbacks and challenges associated with stable expression, including inadequate protein expression, time cost and so on. In this study, we used the vector pCHF3–Flag–Apo A-I_Milano_ to generate a recombination protein in *N. tabacum*. The total protein was extracted from the leaf of 3 day post-infiltration and western blot was performed using Apo A-I monoclonal antibody (Santa Cruze Biotechnology Inc.). The results showed that recombinant protein was produced in *N. tabacum* at approximately 30 kDa (Fig. 6A).

#### Purification and purity of the transient transformation fusion protein Flag–Apo A-I_Milano_

**Fig. 7:**
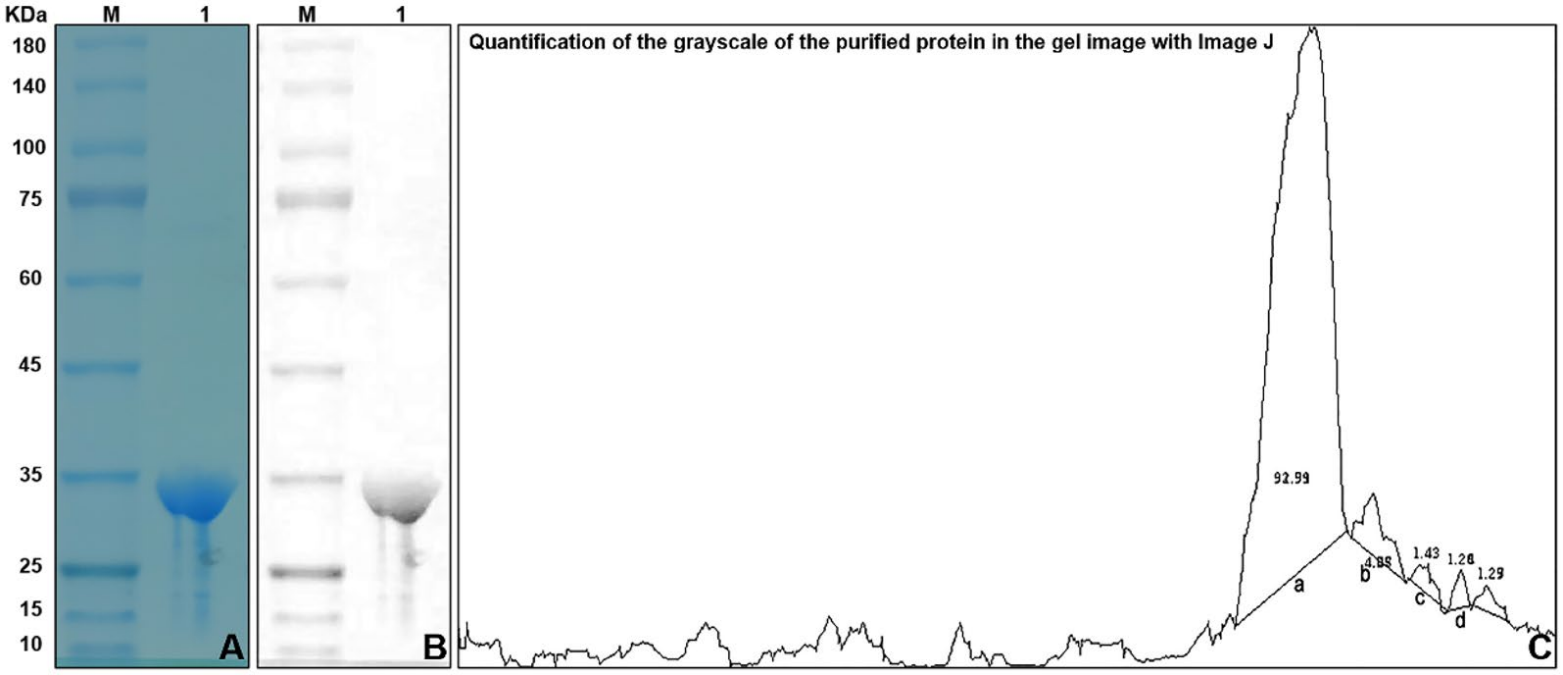
Representative of purification of Flag–Apo A-I_Milano_ protein by SDS–PAGE gel. **A** Flag–Apo A-I_Milano_ purified protein was separated by SDS–PAGE gel electrophoresis and stained with Coomassie brilliant blue. Lane M is standard protein molecular weight marker. Lane 1 presents purified Flag–Apo A-I_Milano_ protein. **B** Image of the SDS–PAGE of Flag–Apo A-I_Milano_ Purified Protein. **C** Quantification of the grayscale of the purified protein in the gel image with Image J. The ratio of the purified Flag–Apo A-I_Milano_ to the total protein. The purity of Flag–Apo A-I_Milano_ calculated by three experiments is 90.58% ± 1.65

The Flag–Apo A-I_Milano_ protein was purified using proteinA/G agarose. Western blot results showed that the purified protein solution had only one clear band, and the size was consistent with the expected theoretical value (30 KDa), indicating that the target protein was purified to a higher degree. The protein concentration was determined by the bicinchoninic acid (BCA) method, according to the standard protein curve, the protein concentration of Flag–Apo A-I_Milano_ after purification was calculated to be 0.84 mg/ml, a total of 0.4 mg (Fig. 6B). Coomassie blue staining of recombinant Flag–Apo A-I_Milano_ proteins in SDS–PAGE (Fig. 6C). SDS–PAGE of Flag–Apo A-I_Milano_ Purified Protein (Fig. 7A), Image of the SDS–PAGE of Flag–Apo A-I_Milano_ Purified Protein (Fig. 7B), Quantification of the grayscale of the purified protein in the gel image with Image J (Fig. 7C).

#### Subcellular localization of Apo A-I_Milano_ in *N. benthamiana* by confocal laser microscopy

**Fig. 8:**
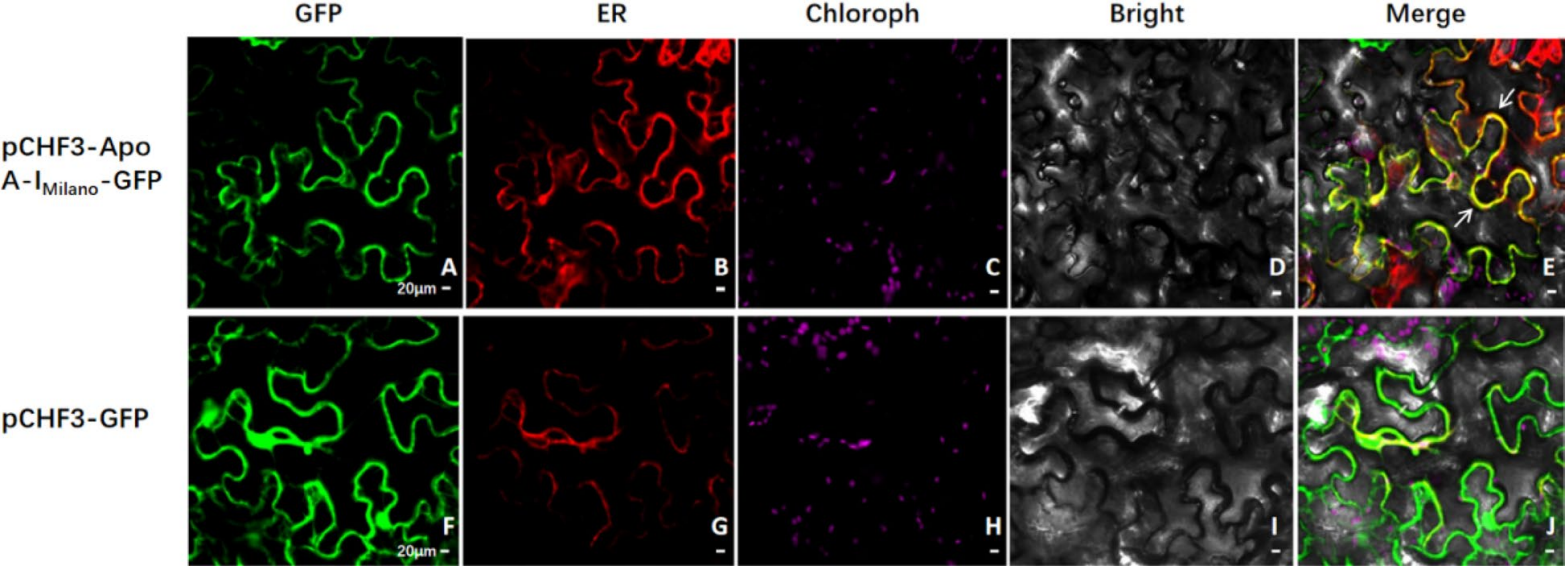
Subcellular co-localization of pCHF3–Apo A-I_Milano_–GFP protein with compartmental markers in *N. benthamiana* (40X). **A**–**E** GFP fluorescence of Apo A-I_Milano_–GFP Fusion protein transiently expressed in *N. benthamiana*; endoplasmic reticulum fluorescence (sper, Red); chlorophyll fluorescence (Purple); bright field and merged; subcellular co-localization of pCHF3–GFP in *N. benthamiana* with compartmental markers. **F**–**J** GFP fluorescence; endoplasmic reticulum fluorescence (sper, Red); chlorophyll fluorescence (Purple); bright field and merged. The arrows point out co-localization events. Scale bar: 20 µm

To analyze the subcellular localization of Apo A-I_Milano_, pCHF3–Apo A-I_Milano_–GFP plasmid was constructed, and an empty pCHF3–GFP vector was injected into *N. benthamiana* leaves as the control, respectively, and observed by a laser microscope. It was found that the control group had strong green fluorescence signal in the cells, while pCHF3–Apo A-I_Milano_–GFP had green fluorescence in the endoplasmic reticulum of cells in *N. benthamiana* (Fig. 8), indicating that Apo A-I_Milano_ was located in the endoplasmic reticulum.

#### Determination of amino acid sequence of Apo A-I_Milano_ fused in *N. tabacum*

**Fig. 9:**
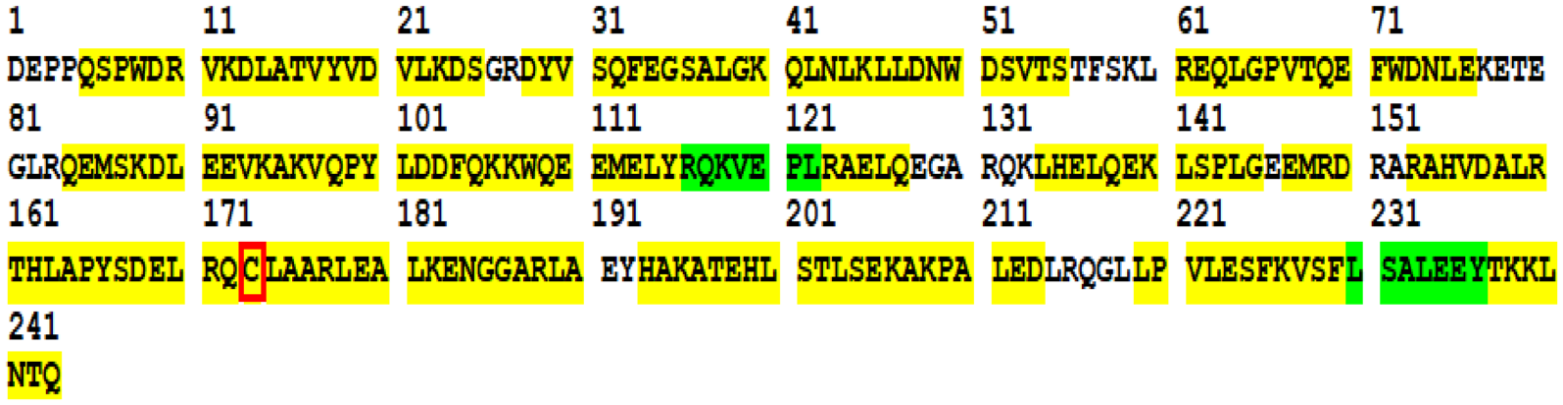
Tandem mass spectrometric coverage of the Apo A-I_Milano_. The representation of Apo A-I_Milano_ amino acid sequence through three sequencing results is shown in Fig. 9. The amino acids contained in each blue line represent the identified amino acids, and several blue lines indicate how many times they have been identified. The amino acids of the Apo A-I_Milano_ present in the fusion protein used in this study showed 86% coverage

To confirm the accuracy of the expression of the target protein in *N. tabacum*, the amino acid sequences of Apo A-I_Milano_ and Flag fusion proteins were determined and analyzed. The peptides were re-dissolved in solvent A (A: 0.1% formic acid in water) and analyzed by Orbitrap Fusion coupled to an EASY-nanoLC 1200 system (Thermo Fisher Scientific, MA, USA). Tandem mass spectra were processed by PEAKS Studio version 10.6 (Bioinformatics Solutions Inc., Waterloo, Canada). The amino acid sequence of the Apo A-I_Milano_ present in the fusion protein is shown in Fig. 9 and yielded 86% coverage, it is also found that Cysteine replaces Arginine at position 173 (marked with red box), which indicates that Apo A-I_Mlano_, a mutant of Apo A-I, is accurately expressed in *N. tabacum*.

#### Activity detection of Flag–Apo A-I_Milano_ protein

**Fig. 10:**
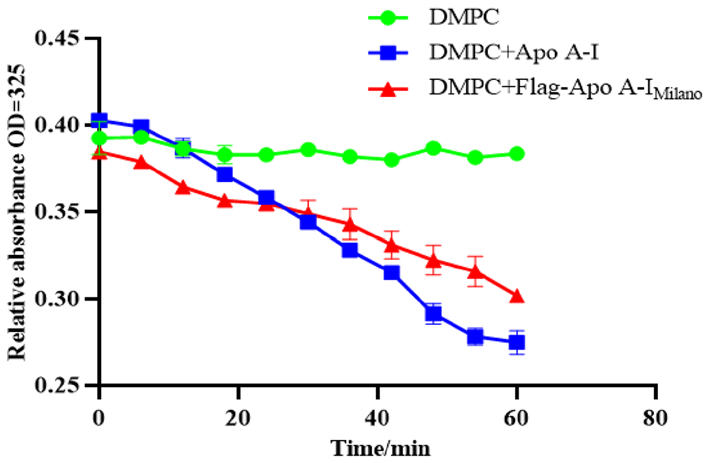
Kinetics of the interaction of Flag–Apo A-I_Milano_ with DMPC. The changes in turbidity were monitored by the change in absorbance at 325 nm at 2 min intervals for the initial 60 min and plotted as a function of time. The DMPC turbidity clearance assay was used to measure the abilities of Flag–Apo A-I_Milano_ protein to combine with lipids. The level of ability was represented by the following: green curve represents the absorbance value of the blank control group, and the blue curve represents the absorbance value of the standard product. The red curve represents the absorbance value of the purified target protein

The activity of Flag–Apo A-I_Milano_ can be determined by dimyristoyl phosphatidyl choline (DMPC) turbidimetric clarification assay, which was used to measure the abilities of Flag–Apo A-I_Milano_ protein to combine with lipids. When Flag–Apo A-I_Milano_ was combined with DMPC, the turbidity of the reaction system decreased. The faster the turbidity decreased, the better the Flag–Apo A-I_Milano_ could bind lipids. As can be seen from Fig. 10, the absorbance value of the blank control group only decreased a little, and the absorbance value of the standard product decreased fastest. The absorbance value of the purified target protein decreased at a rate similar to that of the standard product. Results from this assay showed similar trend in lipid binding activity for both the Flag–Apo A-I_Milano_ sample derived from *N. tabacum* and human Apo A-I protein control.

## Discussion

Pharmaceutical and clinical studies have indicated the potential functions of Apo A-I_Milano_ in reducing atherosclerosis (Ibanez et al. 2008; Nissen et al. 2003; Parolini et al. 2008; L. Wang et al. 2006), preventing restenosis after coronary stenting (Kaul et al. 2003; Speidl et al. 2010), reducing myocardial ischemia (Marchesi et al. 2008) and easing features of Alzheimer’s disease (Fernandez-de Retana et al. 2017).Clinical application of Apo A-I_Milano_ in the future will require a large amount of high quality and cost-effective Apo A-I_Milano_. Thus, various manufacturing systems have been developed. The basic quality of such a system is the ability to express the bioactive target protein. Other superior qualities of an appropriate production system include easily and cheaply for maintenance, cost-efficient, high productivity and the ability for large scale manufacturing.

In generalbioreactors could be classified into microorganism bioreactors, plant-based and animal-based bioreactors. The advantage of animal-based bioreactors is their ability to produce bioactive therapeutic proteins with high human compatibility. However, the cost is quite high for the construction and maintenance of an animal or animal cell bioreactor system (Y. Wang et al. 2013). Microorganism production systems are easy to operate and scale up. However, different translational modifications may lead to the expression of non-soluble and/or non-functional proteins (Swartz 2001). Plant-based bioreactors, such as transgenic plants and plant tissue culture systems, offer cheap and easily scalable production of materials (Xu et al. 2016).

Plant bioreactors can be divided into stable expression systems and transient expression systems according to whether exogenous genes can be stably inherited by offspring. Stable expression systems are the key technology for obtaining stable genetic transgenic plants. Compared with the stable expression system, transient expression does not need to integration foreign genes into the genome, which has the advantages of a short expression cycle and high expression volume (Nosaki et al. 2021), and is an important means for later functional analysis.

*N. tabacum* is a model plant, with advantages, such as easy planting, short growth period, high yield per plant, easy transfer of exogenous genes and mature genetic transformation system, so it can reduce production costs and provide possibilities for large-scale production of exogenous proteins. Therefore, we constructed three expression systems in *N. tabacum* at the same time to confirm the expression of Apo A-I_Milano_ at the mRNA and protein levels, as well as determination of its amino acid sequence. The purpose was to quickly determine the expression characteristics and protein structure and function of Apo A-I_Milano_ through the transient expression system, and to construct a stable expression system of Apo A-I_Milano_ in *N. tabacum*, the purpose was to observe the genetic stability of Apo A-I_Milano_ expression by subculturing Apo A-I_Milano_ positive seeds harvested in a stable expression system. We also designed the fusion expression of Apo A-I_Milano_ and Flag, and obtained the target protein with purity of 90.58% ± 1.65. *N. tabacum* was selected under antibiotic stress. PCR and RT-qPCR were performed to examine the presence of the Apo A-I_Milano_ gene. Moreover, the expression of Apo A-I_Milano_ was analyzed by Western blot.

Humanized proteins and plant derived proteins have great differences in post-translational modification, especially in glycosylation modification. Several literatures have reported glycosylation modification of plant chassis. The glycosylation of humanized proteins and plant derived proteins on endoplasmic reticulum is basically the same, but the glycosylation of proteins located in Golgi matrix is quite different (Schoberer et al. 2018). This study identified the organelle localization of the target protein through subcellular localization. The results showed that the Apo A-I_Milano_ protein was located on the endoplasmic reticulum of *N. bentamiana*. It was speculated that the glycosylation modification of the Apo A-I_Milano_ protein after expression in N. bentamiana should be consistent with that in mammalian cells, providing a basis for the next activity identification. the subcellular localization studies were performed with *N. bentamiana* rather than *N. tabacum* to avoid the huge overexpression in *N. tabacum* that hampers subcellular localization studies, so I chose *N. bentamiana* is used for subcellular localization.

In the experiment, to more accurately clarify the expression of Apo A-I_Milano_ fusion protein in *N. tabacum*, the purified Apo A-I_Milano_ fusion protein was sequenced and identified by tandem mass spectroscopy, according to the preliminary analysis, the amino acid sequence of Apo A-I_Milano_ expressed in *N. tabacum* was compared with that of normal human Apo A-I sequence (274aa, Uniprot/Swiss prot: P02647.1), and the coverage rate was 86% which was analyzed together with the above other identification results, it shows the accuracy and authenticity of the expression of Apo A-I_Milano_ in *N. tabacum*. Next, we further analyzed the post-translational modification of the protein expressing Apo A-I_Milano_ in *N. tabacum*, O- and N-terminal glycosylation and its functional analysis and clinical trials, to further promote the possibility of its clinical application and accelerate the pace of its clinical application. The protein activity and function were analyzed by the DMPC turbidity clarification test. Meanwhile, post-translational modification, and protein function tests of exogenous proteins are underway. Although many scientists have recently done a lot of work on plant bioreactors and achieved unprecedented results, especially in the expression of medicinal proteins, some biopharmaceuticals beneficial to human health have also been discovered, including monoclonal antibodies and vaccines, some progress has also been made in the plant seed expression system (Nykiforuk et al. 2011). However, the expression of Apo A-I_Milanno_ in the model plant *N. tabacum* is the first report. It is currently superior to other expression systems in terms of performance and yield. In addition, although using transgenic plants to produce medical protein provokes some concerns, such as the need to improve the amount of heterologous protein expressed, the doubt about the differences in the method of glycosylation, with the huge market demand and the tireless efforts of scientific research personnel, accompanied by the optimization and extensive use of the system step by step, it will come true using plant bioreactors to produce medical protein.

In conclusion, we presented the establishment of an *N. tabacum* culture system suitable for Apo A-I_Milano_ expression. Our future work will focus on Apo A-I_Milano_ bioactivity characterization. The final aim will be large scale production of bioactive Apo A-I_Milano_. The *N. tabacum* culture system appears to provide a viable, cost-efficient, and environmentally friendly platform for the production of pharmaceutically bioactive proteins.

## Data Availability

The data sets used and/or analyzed are available from the corresponding author on reasonable request.
